# Comparative analysis of the value of amide proton transfer-weighted imaging and diffusion kurtosis imaging in evaluating the histological grade of cervical squamous carcinoma

**DOI:** 10.1186/s12885-022-09205-z

**Published:** 2022-01-20

**Authors:** Mengyan Hou, Kai Song, Jipeng Ren, Kaiyu Wang, Jinxia Guo, Yongchao Niu, Zhenyu Li, Dongming Han

**Affiliations:** 1 Department of MRI, Xin Xiang Central Hospital & The Fourth Clinical College of Xinxiang Medical University, 56 Jinsui Road, Xinxiang, 453000 Henan China; 2 Department of Orthopedics, the First Affiliated Hospital, Xinxiang Medical University, Weihui, China; 3 Department of MRI, the First Affiliated Hospital, Xinxiang Medical University, 88 Jiankang Road, Weihui, 453100 China; 4 MR Research China, GE Healthcare, Beijing, China

**Keywords:** Cervical squamous cell carcinoma, Amide proton transfer-weighted imaging, Diffusion kurtosis imaging, Diffusion-weighted imaging

## Abstract

**Background:**

Uterine cervical cancer (UCC) was the fourth leading cause of cancer death among women worldwide. The conventional MRI hardly revealing the microstructure information. This study aimed to compare the value of amide proton transfer-weighted imaging (APTWI) and diffusion kurtosis imaging (DKI) in evaluating the histological grade of cervical squamous carcinoma (CSC) in addition to routine diffusion-weighted imaging (DWI).

**Methods:**

Forty-six patients with CSC underwent pelvic DKI and APTWI. The magnetization transfer ratio asymmetry (MTRasym), apparent diffusion coefficient (ADC), mean diffusivity (MD) and mean kurtosis (MK) were calculated and compared based on the histological grade. Correlation coefficients between each parameter and histological grade were calculated.

**Results:**

The MTRasym and MK values of grade 1 (G1) were significantly lower than those of grade 2 (G2), and those parameters of G2 were significantly lower than those of grade 3 (G3). The MD and ADC values of G1 were significantly higher than those of G2, and those of G2 were significantly higher than those of G3. MTRasym and MK were both positively correlated with histological grade (r = 0.789 and 0.743, *P* <  0.001), while MD and ADC were both negatively correlated with histological grade (r = − 0.732 and - 0.644, P <  0.001). For the diagnosis of G1 and G2 CSCs, AUC (APTWI+DKI + DWI) > AUC (DKI + DWI) > AUC (APTWI+DKI) > AUC (APTWI+DWI) > AUC (MTRasym) > AUC (MK) > AUC (MD) > AUC (ADC), where the differences between AUC (APTWI+DKI + DWI), AUC (DKI + DWI) and AUC (ADC) were significant. For the diagnosis of G2 and G3 CSCs, AUC (APTWI+DKI + DWI) > AUC (APTWI+DWI) > AUC (APTWI+DKI) > AUC (DKI + DWI) > AUC (MTRasym) > AUC (MK) > AUC (MD > AUC (ADC), where the differences between AUC (APTWI+DKI + DWI), AUC (APTWI+DWI) and AUC (ADC) were significant.

**Conclusion:**

Compared with DWI and DKI, APTWI is more effective in identifying the histological grades of CSC. APTWI is recommended as a supplementary scan to routine DWI in CSCs.

## Introduction

Uterine cervical cancer (UCC) was the fourth most commonly diagnosed malignancy and the fourth leading cause of cancer death among women worldwide in 2018 [1]. Cervical squamous carcinoma (CSC) is the most common pathological type of UCC, accounting for 75–80% of the total number of cervical cancer patients [2]. Poorly differentiated CSCs can easily cause local invasion and distant metastasis, affecting the choice of treatment and prognosis of patients [3, 4]. Therefore, it is important to accurately assess the grade of CSC before treatment. The clinical diagnosis and evaluation of the pathological features of UCC are conducted through puncture biopsy, but the size of lesions, accuracy of sampling, and other factors [5] tend to cause differences between the results and the final pathology. Therefore, imaging methods are used as a complement for CSC grading.

Magnetic resonance imaging (MRI) has the characteristics of high-resolution soft tissue and multidirectional imaging [6, 7] and plays an important role in the staging and evaluation of cervical cancer. However, the conventional MRI scan sequence can only reflect the anatomical features of soft tissue, hardly revealing the microstructure information. Diffusion models, such as Gaussian distribution-based diffusion-weighted imaging (DWI) and non-Gaussian distribution-based diffusion kurtosis imaging (DKI) [8, 9], can noninvasively detect the diffusion motion of water molecules in living tissue and reflect changes in biological microstructure. Several studies have reported the utility of DWI in predicting the histologic type and tumor recurrence of UCC [10, 11]. However, its value in identifying the pathological grade of CSC is still controversial. DKI, a new MR technology developed based on DWI, can more accurately reflect the complexity of organizational microscopic environments. The clinical application of DKI in evaluating the grade of gliomas and prostate cancers [12–14] has been reported. Wang et al. [15] reported that DKI based on the non-Gaussian diffusion model can be used to distinguish the stage and grade of UCC according to a pilot study. Amide proton transfer-weighted imaging (APTWI) is a molecular imaging technology developed based on chemical saturation transfer (CEST) that can noninvasively detect the exchange process between amide protons and water molecules, revealing information on cell metabolism and pathophysiology [16–18]. APTWI shows clinical application value in evaluating the pathological grading of brain tumors, prostate cancer, and endometrial cancer [19–21]. Preliminary studies [22–24] have shown that APTWI can be used to diagnose and predict the pathological type of UCC and evaluate the histological grade of CSC, providing certain reference values for clinical diagnosis and treatment decisions.

The purpose of this study was to compare the value of APTWI and different diffusion models (DWI, DKI) in differentiating the histological grades of CSCs. In particular, in addition to routine DWI, these two techniques are more suitable for future CSC diagnosis.

## Materials and methods

### Patients

**Fig. 1:**
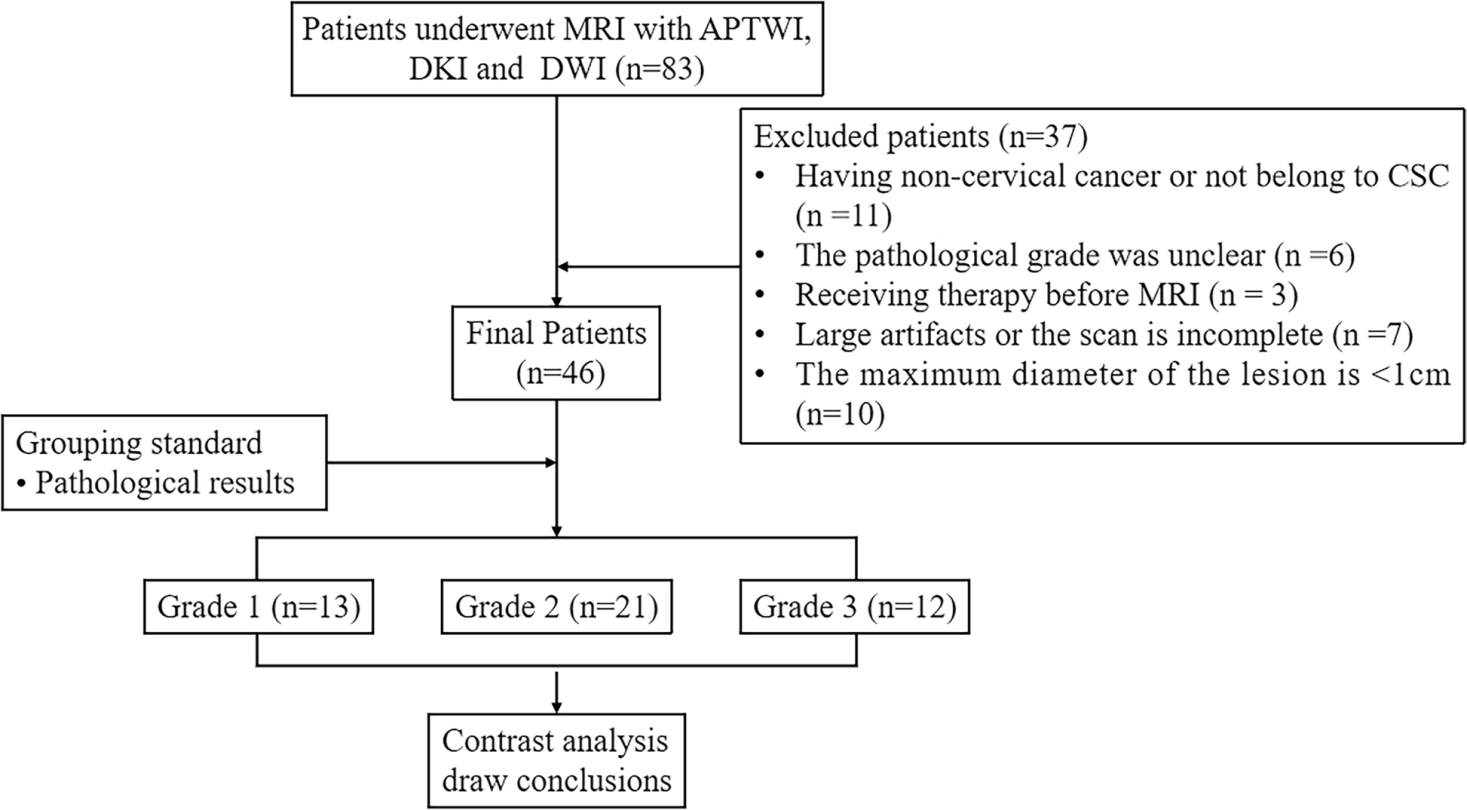
Flow diagram of the patient selection process

This prospective study was approved by the ethics committee of the hospital, and all subjects signed an informed consent form before the examination. From June 2017 to March 2019, a consecutive series of 83 female patients were enrolled for pelvic MRI in this study due to suspicion of EC according to computed tomography (CT) or ultrasound (US). The exclusion criteria were as follows: 1) pathological results showed cervical adenocarcinoma or did not meet the diagnosis of cervical cancer (*n* = 11); 2) clinical results were consistent with CSC, but the pathological grade was unclear (*n* = 6); 3) radiotherapy, chemotherapy, or medication were applied before MRI (*n* = 3); 4) there were large artifacts in the scanned image or the scan was incomplete. (*n* = 7); and 5) the maximum diameter of the lesion was < 1 cm (*n* = 10). Ultimately, a total of 46 patients with CSC were included (aged 35 ~ 70 years). A summary flowchart is presented in Fig. 1.

### MRI technique

Data were acquired on a 3.0 T MRI scanner (Discovery MR750, GE Healthcare, Milwaukee, WI) with a 32-channel body phased-array coil. Before the examination, the patient’s bladder and vagina were moderately filled with bladder and vaginal tamponade for better observation and scanning of lesions. The examination ranging from the anterior superior iliac spine to the pubic symphysis were sequentially taken with conventional MRI, DWI, DKI, and APTWI. Patients did not receive any form of enhanced imaging 24 h before APTWI examination to avoid interference with the APTWI signals. Parameters details for each sequence are shown in Table 1.

**Table 1:** Imaging protocol parameters

Parameters	T1WI	T2WI	DWI	DKI	APTWI
Sequence	FSE	FSE	SS-EPI	SS-EPI	EPI
Orientation	Axial	Axial	Axial	Axial	Axial
FOV (cm^2^)	36 × 36	36 × 36	36 × 36	36 × 36	36 × 36
Matrix	320 × 224	320 × 224	128 × 128	128 × 128	128 × 128
TR/TE (ms)	605/8	5455/109	6000/60.5	2500/58.9	3000/12
Slice thickness	5	5	5	5	5
Slice gap (mm) (mm)	1	1	1	1	1
NEX	1	1	1, 4	2	1
b-values (s/mm^2^)	/	/	0, 800	0, 500,1000,1500,2000	/
saturation pulse/time	/	/	/	/	2.0 μT, 500 ms
Frequency list (only APTWI)	52 frequencies in total: 5000, 5000, 5000, ±600, ±575, ±550, ±525, ±500, ±475, ±450, ±425, ±400, ±375, ±350, ±325, ±300, ±275, ±250, ±225, ±200, ±175, ±150, ±125, ±100, ±75, ±50, ±25 Hz				
Scan time	1 min 57 s	1 min 33 s	1 min 24 s	5 min 28 s	2 min 36 s (Single layer)

*FSE* fast spin echo, *SS-EPI* single Shot Echo Planar Imaging, *TR/TE* repetition time/echo time, *FOV* field of view, *NEX* number of excitations. The number of DKI diffusion gradient directions is 30

### Postprocessing and analysis

The DWI, DKI, and APTWI images were transferred to a workstation (Advantage Workstation 4.6, GE Healthcare) for postprocessing. The formula of the DWI model is as follows:

$${\mathrm{S}}_{\left(\mathrm{b}\right)}={\mathrm{S}}_0\times \exp \left(-\mathrm{b}\times \mathrm{ADC}\right)$$

where S0 refers to the signal intensity (SI) without the diffusion gradient applied, S(b) refers to the SI when the diffusion gradient is applied, and the b value refers to the diffusion weight [25]. The formula of the DKI model is as follows:

$${\mathrm{S}}_{\left(\mathrm{b}\right)}={\mathrm{S}}_0\times \exp \left(-\mathrm{b}\times MD+{\mathrm{b}}^2\times {MD}^2\times MK/6\right)$$

where mean diffusivity (MD) represents the apparent diffusion coefficient (ADC) after correction and mean kurtosis (MK) represents the degree to which the dispersion deviates from the Gaussian distribution [26]. The SI of APTWI can be obtained by measuring the magnetization transfer rate by calculating the difference in SI at ±3.5 ppm on both sides of the water center frequency in the Z-spectrum. The formula is as follows:

$$\mathrm{MTRasym}\kern0.5em \left(3.5\mathrm{ppm}\right)=\left[{\mathrm{S}}_{\mathrm{sat}}\left(-3.5\mathrm{ppm}\right)-{\mathrm{S}}_{\mathrm{sat}}\left(+3.5\mathrm{ppm}\right)\right]/{\mathrm{S}}_0$$

where MTRasym (or magnetization transfer ratio asymmetry) (3.5 ppm) is the asymmetric magnetization transfer rate at 3.5 ppm downfield from the water signal, So is the SI without the saturation pulse applied, and S_sat_ is the SI after the saturation pulse is applied [27].

While blinded to the postoperative pathology, two radiologists with 5 and 10 years of experience in MR diagnosis independently completed the measurement of the DWI, DKI and APTWI parameters, including ADC, MK, MD, and MTRasym values. Conventional T1WI, T2WI, and DWI images were used to determine the parenchymal portion of the tumor. The regions of interest (ROIs), excluding areas with necrosis, obvious signals or artifacts from a hemorrhage, cystic degeneration, and blood vessels, were drawn along the edge of the parenchymal portion of the tumor at all slices of the tumor tissue. The final value of each lesion parameter was the average value of the corresponding parameter on all slices. The calculation formula of tumor volume based on T2WI images is as follows: tumor volume = the sum of the tumor area of each slice × (slice thickness + interslice gap).

### Histopathologic analysis

A pathologist (with 8 years of experience) who was blinded to the MRI data analyzed all surgically resected specimens of each patient. Hematoxylin/eosin (HE) staining was used to determine the histological grade. With reference to the International Federation of Gynecology and Obstetrics (FIGO) grading system [23], the specimens were classified into grade 1 (G1, *n* = 13), grade 2 (G2, *n* = 21) and grade 3 (G3, *n* = 12) groups.

### Statistical analysis

MedCalc (Version 15.0; MedCalc Software) and SPSS (Version 23.0; IBM) software were used for statistical analysis. Intraclass correlation coefficients (ICCs) were used to evaluate the consistency of the results calculated by 2 experienced radiologists (r ≥ 0.75, excellent; 0.60 ≤ r <  0.75, good; 0.40 ≤ r <  0.60, fair; and r <  0.40, poor). The Shapiro-Wilk test was used to evaluate whether the measurements were normally distributed. The obtained parameters were compared by one-way analysis of variance with Bonferroni’s honestly significant difference post hoc test. Receiver operating characteristic (ROC) analysis was used to evaluate the diagnostic performance of the obtained parameters, and the Delong test was utilized to determine whether the area under the ROC curve (AUC) of each parameter was different. Spearman correlation analysis was performed to analyze the correlation between the parameters and the grade differentiation of CSCs (r ≥ 0.75, good; 0.50 ≤ r <  0.75, moderate; 0.25 ≤ r <  0.50, mild; and r <  0.25, little or none). *P* <  0.05 was considered statistically significant.

## Results

### Interobserver agreement

**Fig. 2:**
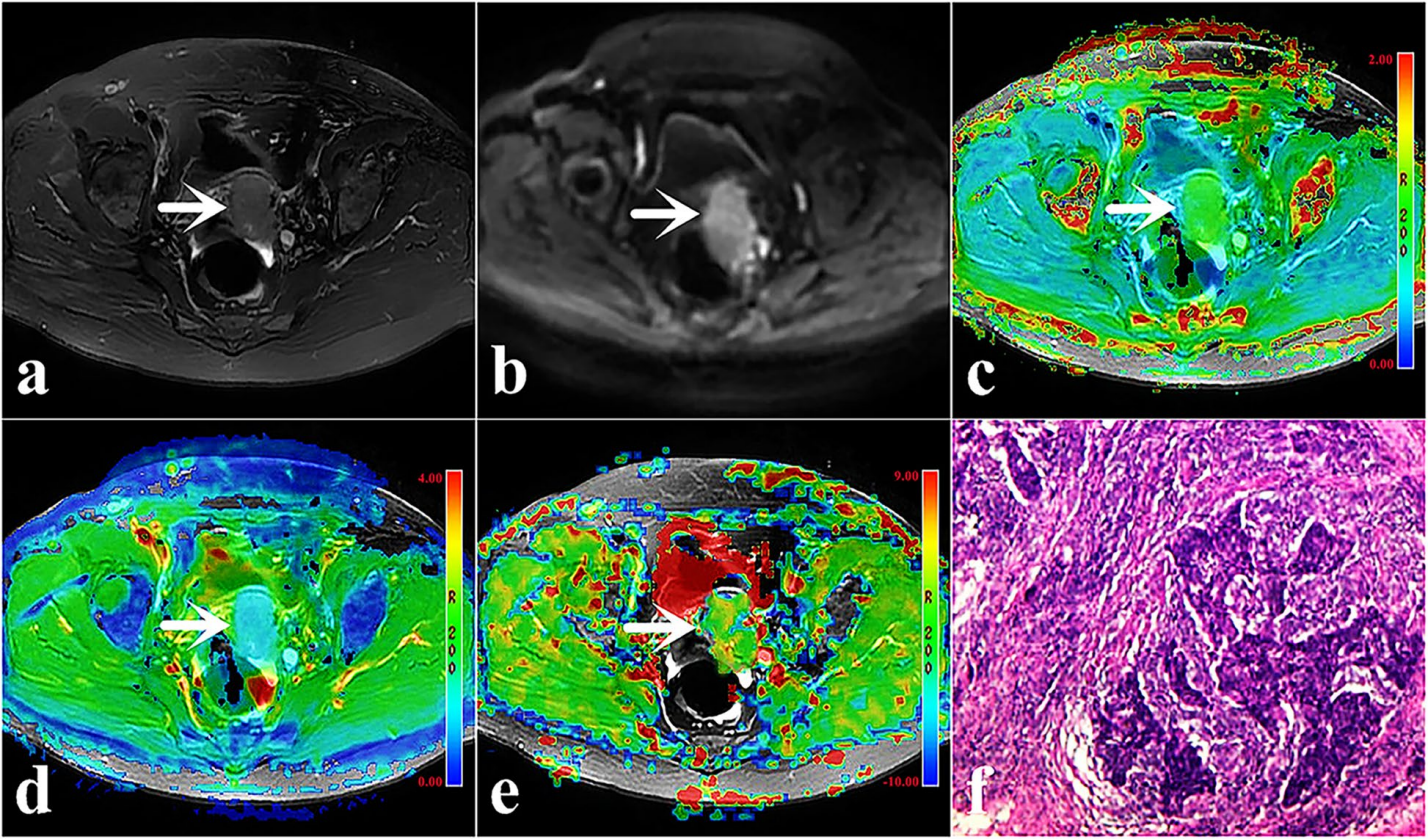
Grade 3 of CSC in a 42-year-old woman (arrowheads), ADC = 0.94 × 10^− 3^/mm^2^, MK = 0.90, MD = 1.03 × 10^− 3^/mm^2^, and MTRasym = 3.07%. **a** Map of T2WI, **b** Map of DWI (b = 1000 s/mm^2^), **c** Pseudo colored maps of MK, **d** Pseudo colored maps of MD, **e** Pseudo colored maps of MTRasym, **f** Pathological images (original magnification, × 100)

The MTRasym, MK, MD, and ADC values measured by the two observers had an excellent agreement (*P* <  0.001), with ICC values of 0.85, 0.86, 0.78, and 0.90, respectively. Therefore, we chose the averages of the parameter values measured by the 2 observers for further analysis. The original images generated by DWI, DKI, and APTWI and maps derived from the data are shown in Fig. 2.

### The value of parameter changes in the three CSC grades

**Fig. 3:**
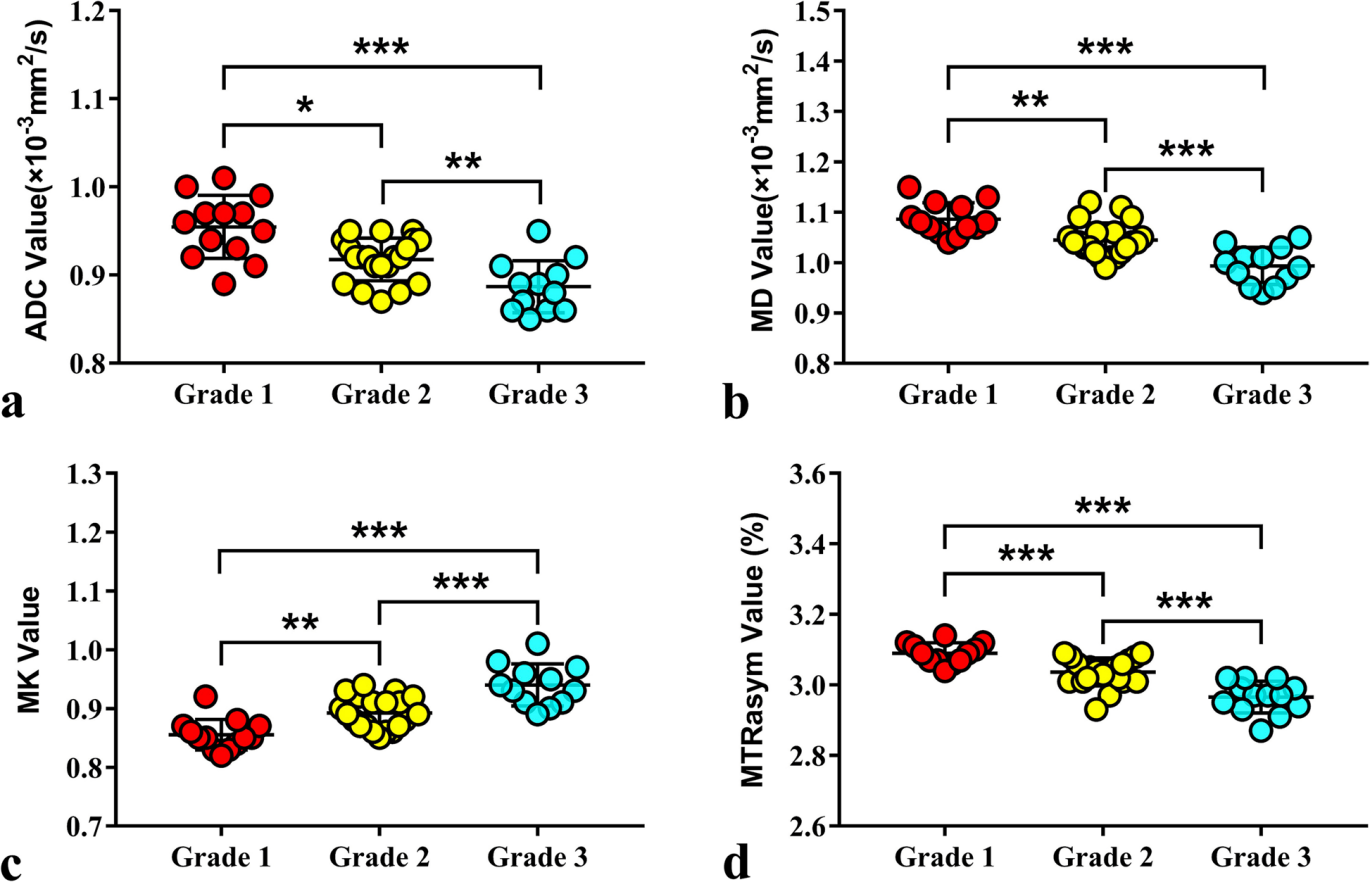
Plots show individual data points, averages, and standard deviations of ADC (**a**), MD (**b**), MK (**c**), and MTRasym (**d**) in different groups. Individual points are averages of values calculated by 2 readers. *P <  0.05, **P <  0.01, and ***P <  0.001

**Fig. 4:**
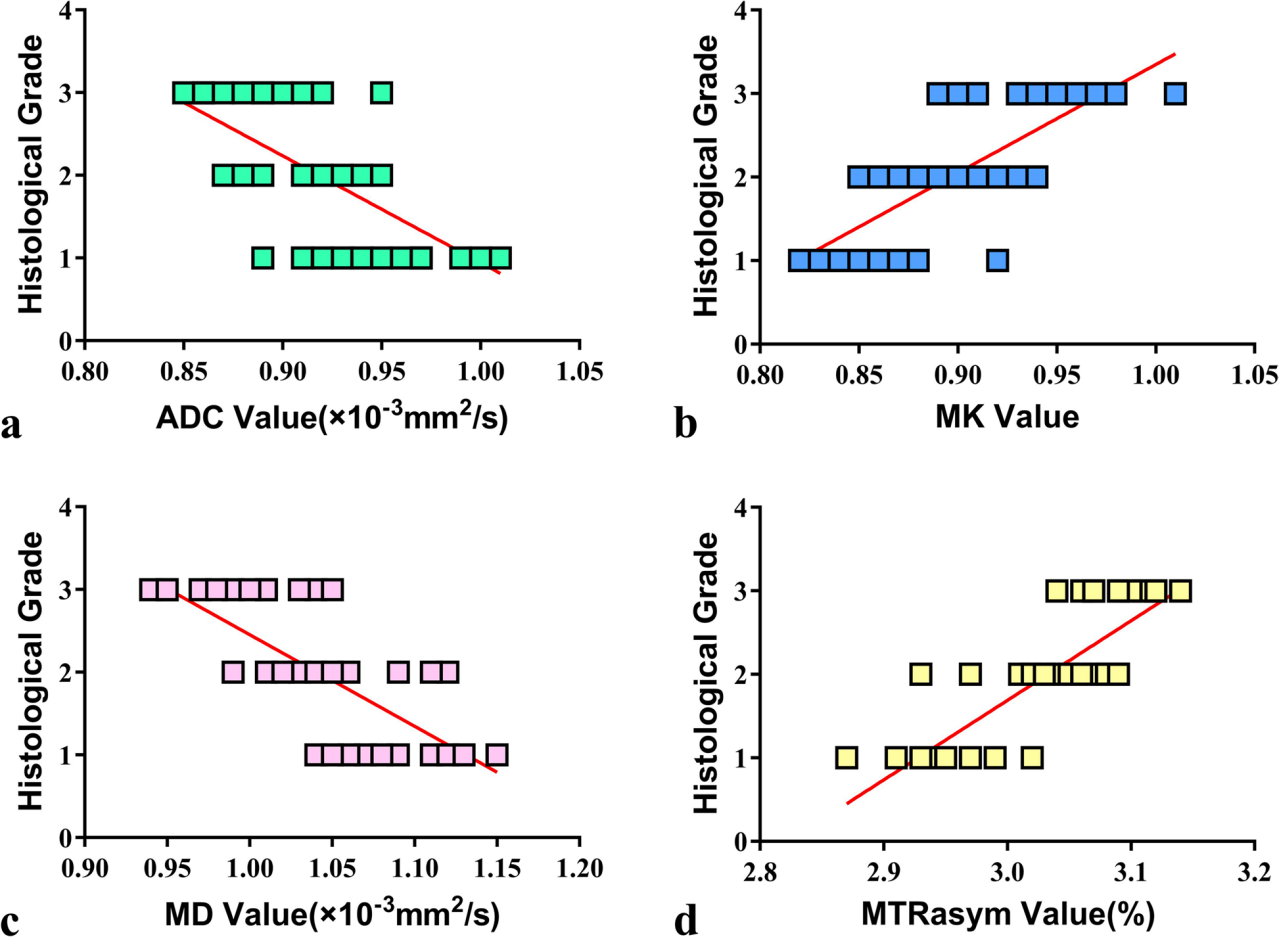
The correlation between histological grading and different parameters. The ADC (**a**) and MD (**c**) are also well correlated with grades (r = − 0.644, − 0.732, P <  0.001), while MTRasym (**d**) and MK (**b**) are strongly correlated with pathological grade (r = 0.789, 0.743, P <  0.001)

The MTRasym and MK values of grade 1 were significantly lower than those of G2, and those parameters of G2 were significantly lower than those of G3 (G1 < G2 < G3, all *P* <  0.001). The MD and ADC values of G1 were significantly higher than those of G2, and those parameters of G2 were significantly higher than those of G3 (G1 > G2 > G3, all P <  0.001). MTRasym and MK were both positively correlated with histological grade (r = 0.789 and 0.743, P <  0.001), while MD and ADC were both negatively correlated with histological grade (r = − 0.732 and - 0.644, P <  0.001). The details are shown in Table 2 and Figs. 3 and 4.

**Table 2:** Comparisons of MTRasym, MK, MD and ADC Among Three Histologic Grades

Parameters	Grade1	Grade2	Grade3	F-value	*P*-value	*P*-value (Grade 1vs.2)	*P*-value (Grade 1vs.3)	*P*-value (Grade 2 vs.3)
Volume (cm^3^)	46.46 ± 17.14	54.24 ± 12.37	54.42 ± 15.61	1.331	0.275	0.422	0.549	0.973
MTRasym (%)	2.96 ± 0.04	3.03 ± 0.04	3.09 ± 0.03	16.974	< 0.001	< 0.001	< 0.001	0.001
MK	0.85 ± 0.03	0.89 ± 0.03	0.94 ± 0.04	26.402	< 0.001	0.002	< 0.001	< 0.001
MD (×10^−3^ mm^2^/s)	1.08 ± 0.03	1.04 ± 0.03	0.99 ± 0.04	22.938	< 0.001	0.004	< 0.001	< 0.001
ADC (× 10^− 3^ mm^2^/s)	0.95 ± 0.04	0.91 ± 0.02	0.88 ± 0.02	32.354	< 0.001	0.003	< 0.001	0.016

### ROC analysis

**Fig. 5:**
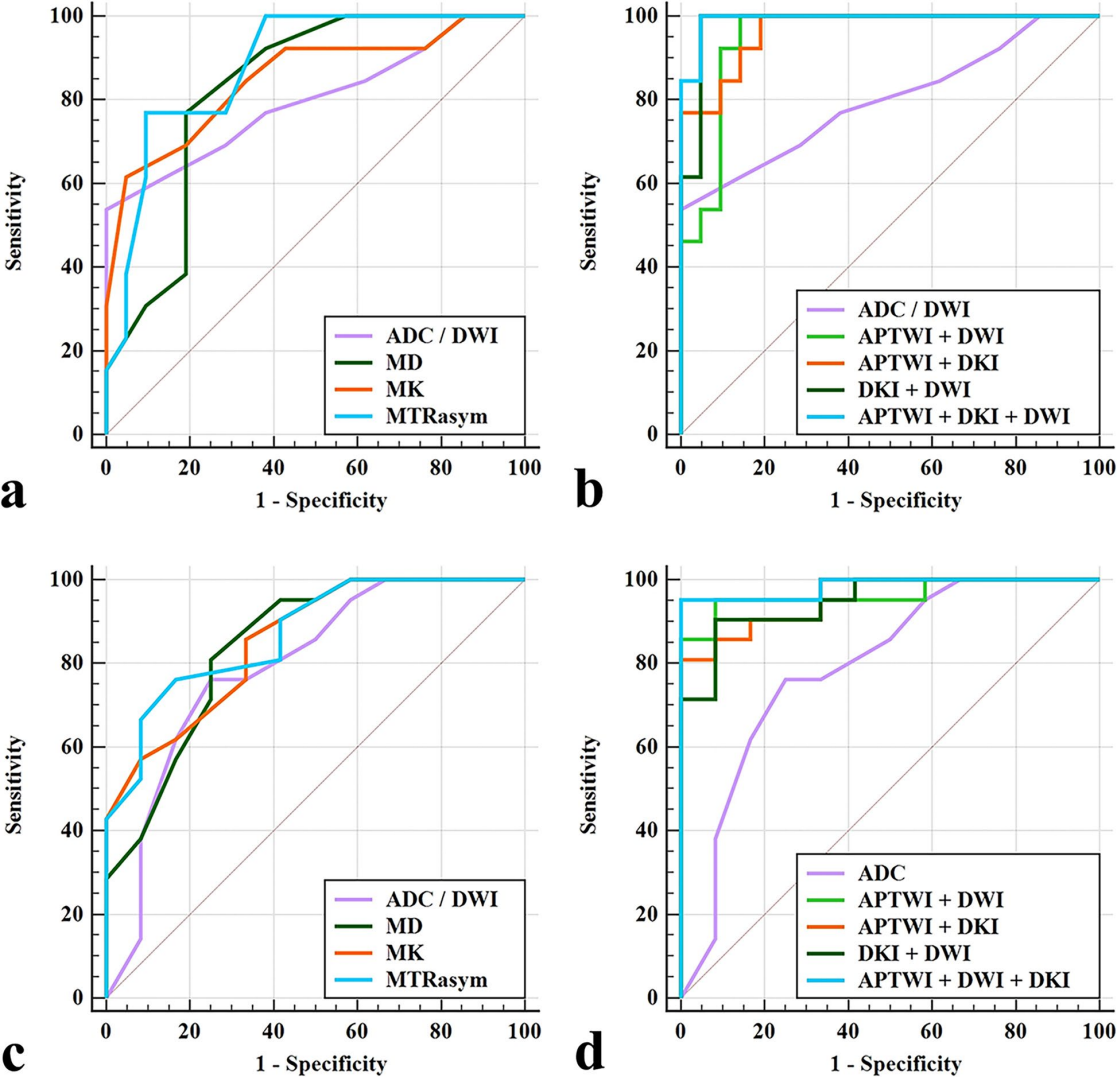
Curves show MTRasym, ADC, MD, MK, APTWI+DWI, APTWI+DKI, DWI + DKI, and APTWI+DWI + DKI by using ROC analysis for differentiation of different groups. Details of the area under the curves and 95% CIs of each index are shown in the Results section and Tables 3 and 4

For the diagnosis of G1 and G2 CSCs, AUC (APTWI+DKI + DWI) > AUC (DKI + DWI) > AUC (APTWI+DKI) > AUC (APTWI+DWI) > AUC (MTRasym) > AUC (MK) > AUC (MD) > AUC (ADC), where the differences between AUC (APTWI+DKI + DWI), AUC (DKI + DWI) and AUC (ADC) were significant (Z = 2.282, 2.230; *P* = 0.023, 0.026, respectively). For the diagnosis of G2 and G3 CSCs, AUC (APTWI+DKI + DWI) > AUC (APTWI+DWI) > AUC (APTWI+DKI) > AUC (DKI + DWI) > AUC (MTRasym) > AUC (MK) > AUC (MD > AUC (ADC), where the differences between AUC (APTWI+DKI + DWI), AUC (APTWI+DWI) and AUC (ADC) were significant (Z = 2.278, 2.004; P = 0.023, 0.045, respectively), (Tables 3 and 4, Fig. 5).

**Table 3:** Comparison of ROC curve between Grade 1 and Grade 2 CSC

Parameters	AUC	Threshold	P-value	Sensitivity(%)	Specificity (%)	95% CI
MTRasym (%)	0.883	3.000	< 0.001	76.9%	90.5%	0.726–0.967
MK	0.852	0.875	0.001	84.6%	66.7%	0.688–0.950
MD (×10^−3^ mm^2^/s)	0.828	1.065	0.002	76.9%	81.0%	0.660–0.935
ADC (×10^−3^ mm^2^/s)	0.799	0.955	0.004	53.8%	100%	0.626–0.916
APTWI+DWI	0.949	/	< 0.001	90.5%	92.3%	0.814–0.995
DKI + DWI	0.982	/	< 0.001	100.0%	95.2%	0.865–1.000
DKI + APTW	0.967	/	< 0.001	100.0%	80.95%	0.841–0.999
APTWI+DKI + DWI	0.993	/	< 0.001	100.0%	95.2%	0.884–1.000

APTWI + DWI = MTRasym +ADC; DKI + APTW = MD + MK + MTRasym; DKI + DWI = MD + MK + ADC; APTWI + DKI + DWI = MTRasym + MD + MK + ADC

**Table 4:** Comparison of ROC curve between Grade 2 and Grade 3 CSC

Parameters	AUC	Threshold	P-value	Sensitivity(%)	Specificity (%)	95% CI
MTRasym (%)	0.871	3.065	< 0.001	76.2%	83.3%	0.708–0.962
MK	0.855	0.925	0.001	85.7%	66.7%	0.689–0.953
MD (× 10^− 3^ mm^2^/s)	0.845	1.015	0.001	81.0%	75.0%	0.677–0.947
ADC (×10^−3^ mm^2^/s)	0.794	0.905	0.006	76.2%	75.0%	0.617–0.914
APTWI+DWI	0.964	/	< 0.001	91.7%	95.2%	0.834–0.999
DKI + DWI	0.948	/	< 0.001	90.5%	91.7%	0.811–0.995
DKI + APTWI	0.956	/	< 0.001	80.95%	100.0%	0.822–0.997
APTWI+DKI + DWI	0.984	/	< 0.001	95.2%	100.0%	0.866–1.000

APTWI + DWI = MTRasym +ADC; DKI + APTW = MD + MK + MTRasym; DKI + DWI = MD + MK + ADC; APTWI + DKI + DWI = MTRasym + MD + MK + ADC

## Discussion

### The value of diffusion techniques in the diagnosis of CSC grades

In this study, we found that the MK values of the G2 and G3 CSC groups were higher than those of the G1 and G2 CSC groups (*P* <  0.05), while the MD and ADC values of the G2 and G3 CSC groups were lower than those of the G1 and G2 CSC groups (P <  0.05), respectively. These results are consistent with those of previous studies [8, 15], indicating that DKI and DWI have positive value for the preliminary assessment of the pathological grade of CSCs for the following reasons. MK is related to the heterogeneity of the diffusion environment [28], which means that the more heterogeneous the microenvironment of the tissue is, the greater the MK value. MD and ADC can detect the degree of blocking of water diffusion in the microenvironment of the tissue, which means that the greater the value is, the lower the degree of restriction of water diffusion. Compared with the G1 and G2 CSC groups, the G2 and G3 CSC groups often exhibit a more compact tissue structure, more significant cellular atypia, and greater tissue necrosis [29]. These features increase the complexity of the microstructure of diseased tissues and further restrict the diffusive motion of water molecules, resulting in higher MK values and lower MD and ADC values for the G2 and G3 CSC groups than for the G1 and G2 CSC groups.

### The value of APTWI in the diagnosis of CSC grades

In the comparison of APTWI in different grade groups, we found that the MTRasym values of the G1 and G2 CSC groups were significantly lower than those of the G2 and G3 CSC groups, respectively, which is similar to the findings of previous studies [23, 24], indicating that APTWI has positive value for the preliminary assessment of the pathological grading of CSC. Possible reasons for this finding are as follows. The role of APTWI in tumors is primarily correlated with the tissue contents of mobile proteins and peptides [30, 31]. According to previous literature, an increase in cellular density, nuclear atypia, microvessel density (MVD), and microscopic necrosis can increase the contents of mobile proteins and peptides in tissues [32, 33] Regarding CSCs, compared with low-grade tumors, high-grade tumors usually have a higher cellular density, more significant nuclear atypia, greater MVD, and more microscopic necrosis [34]. Therefore, high-grade tumors have a higher MTRasym.

### Diagnostic efficacy analysis

The AUCs of MK and MD were higher than that of ADC in differentiating grades, suggesting that the DKI model has an advantage over the traditional DWI model in evaluating the histological grade of CSCs, which is similar to the findings of previous studies [15]. The traditional DWI model is based on the assumption that water molecule diffusion in the tissue is subjected to a Gaussian distribution [35], which makes it difficult to accurately reflect the true movement of water molecules in diseased tissue. However, DKI is based on a non-Gaussian distribution model, which can reflect the microstructure information in terms of the degree to which the dispersion of water molecules deviates from the Gaussian distribution [10]. Therefore, DKI can more accurately reflect the microstructure information of the organization, and the MD value derived from DKI refers to the diffusion coefficient after modeling modification, showing a higher accuracy. Yue et al. [36] also found that DKI was superior to conventional DWI in the classification of endometrial cancer and could more effectively evaluate the pathological and physiological characteristics of endometrial cancer.

Among all the parameters in this study, MTRasym showed the highest differential diagnostic efficiency in CSC grading, indicating that APTWI can reflect the microscopic features of tumors better than diffusion imaging models. The possible reasons for this are discussed below. First, the change in diffusion was greater than the change in free protein/polypeptide content in different CSC grades. Second, there are some modeling imperfections of DKI, such as high b values (2000 s/mm^2^) affecting the signal-to-noise ratio (SNR) [37] and limited directions in detection [38], which may lead to deviation in the measurement, while APTWI imaging, based on the detection of endogenous protein and peptide, is not affected by the above factors.

The AUCs of the combination of APTWI and DWI, the combination of DKI and DWI, and the combination of APTWI, DKI, and DWI were all higher than that of DWI. DWI is important for CSC diagnosis and is commonly used as a routine scan sequence. From our results, adding APTWI, DKI, or both to DWI scans may, to varying degrees, improve the diagnostic accuracy in evaluating the histological grade of CSCs. For clinical usage, considering the scanning time, we recommend APTWI as the first choice for supplementary scans of routine DWI in CSC detection. If time permits, users can also add both DKI and APTWI scans.

There are some limitations of this study. 1) Both the DKI and APT sequences we used were based on echo planar (EPI) acquisition, which is susceptible to motion, metal, and air artifacts and subjected to low SNR and distortions, leading to low-quality images for some small lesions, which may affect the accuracy of this experiment to some extent. 2) The optimal b value of DKI and DWI remains to be explored since a publicly recognized standard has not yet been introduced. 3) The manually selected ROI avoided cystic and necrotic tissue areas while reducing the heterogeneity of tumor tissue, affecting the accuracy of some parameters. In the future, we will seek solutions to further improve the accuracy of parameter measurement.

## Conclusion

APTWI and DKI can be used in grading CSC. Compared with DWI and DKI, APTWI is more effective in identifying the histological grades of CSCs. For clinical usage, in addition to routine DWI, APTWI is recommended as the first choice for supplementary scans in CSC detection in the future when pursuing higher diagnostic accuracy.

## Data Availability

The datasets used and/or analysed during the current study available from the corresponding author on reasonable request.
